# Description of clinical and histopathological characteristics of keloids in a sub-Saharan African population

**DOI:** 10.1093/skinhd/vzaf034

**Published:** 2025-07-08

**Authors:** Ronald Mbiine, Misaki Wayengera, Noah Kiwanuka, Ian G Munabi, Haruna Muwonge, Cephas Nakanwagi, Biratu Olika, Lekuya Herve Monka, Moses Joloba, Moses Galukande

**Affiliations:** Department of Surgery, College of Health Sciences, Makerere University, Kampala, Uganda; Department of Immunology and Molecular Biology, School of Biomedical Sciences, College of Health Sciences Makerere University, Kampala, Uganda; School of Public Health, Makerere University College of Health Sciences, Kampala, Uganda; Department of Human Anatomy, School of Biomedical Sciences, Makerere University College of Health Science, Kampala, Uganda; Department of Physiology, Makerere University College of Health Sciences, Kampala, Uganda; Department of Nursing, Mulago National Referral Hospital, Kampala, Uganda; Department of Pathology, Gammapath Clinical Laboratories, Kampala, Uganda; Department of Surgery, College of Health Sciences, Makerere University, Kampala, Uganda; Department of Immunology and Molecular Biology, School of Biomedical Sciences, College of Health Sciences Makerere University, Kampala, Uganda; Department of Surgery, College of Health Sciences, Makerere University, Kampala, Uganda

## Abstract

**Background:**

Keloids are among the leading benign skin conditions globally, affecting up to 16% of the African population. They are characterized by aggressive behaviour, high recurrence rates and poor treatment outcomes, particularly in African populations. Despite this, most information on keloids in sub-Saharan Africa remains anecdotal, with limited comprehensive studies describing their clinical and pathological characteristics.

**Objectives:**

To analyse the clinical and histopathological features of keloids in a sub-Saharan African population.

**Methods:**

Following ethical approval, 231 participants were enrolled in a cross-sectional study conducted at the 2 national referral plastic surgical centres in Uganda. Data on demographics, clinical presentations and histopathological features of keloids were collected and analysed.

**Results:**

Sixty-four per cent of participants (*n* = 148/231) were women, 63.3% (*n* = 133/210) resided in central Uganda and 82.3% (*n* = 190/231) belonged to the Bantu ethnic group, with those belonging to the Baganda tribe predominating (*n* = 97/231; 42.0%). Keloids were broadly classified into sessile (*n* = 116/231; 50.2%) and nodular types. The most frequently affected anatomical sites were the head and neck (*n* = 106/231; 45.9%) and the trunk (*n* = 52/231; 22.5%). Additionally, 58.0% (*n* = 134/231) of participants had multiple keloids. Histopathological analysis of 35 excised specimens revealed that keloids are primarily a dermal disease, characterized by excessive collagen deposition, particularly hyalinized collagen.

**Conclusions:**

Keloids in African populations exhibit a slight female predominance, a tendency toward multiplicity and larger lesion volumes. Histopathological findings highlight excessive collagen deposition as a hallmark, emphasizing the need for tailored approaches to improve treatment outcomes.

## Teaser

Keloids are one of the most common benign skin disorders globally. Despite being described to have high recurrence rates and poor treatment outcomes among Africans, there are limited descriptive data on the clinical and histopathological characteristics among this population. We described these characteristics in an African population and found that these keloids were large and multiple, with extensive keloid collagen deposition.

What is already known about this topic?

- Most often, teaching on keloids is based on Western literature; the absence of the sub-Saharan African context, despite this being the most affected population, has resulted in a knowledge gap.

What does this study add?

- This study provides a baseline description of keloid characteristics in a sub-Saharan African population.
- We found that half of individuals with keloids had multiple keloids.
- The histopathological characteristics of keloids did not differ from those described in non-African keloids.

Keloids are benign fibroproliferative disorders that manifest along a clinical spectrum, ranging from a single mass to extensive multiple scar swellings. Keloids are estimated to affect between 4% and 16% of African people living in sub-­Saharan Africa,^1–3^ which is significantly higher than the prevalence rate in White and other populations, in whom the estimated prevalence rate is approximately 1%.^4,5^ The distribution of keloids varies, often occurring in high-tension areas such as the presternal region and the face. Multiple keloids commonly develop even after minor trauma and are frequently associated with genetic susceptibility. Patients with multiple keloids tend to experience more severe disease manifestations and are at a higher risk of recurrence.^6,7^

Clinically, keloids exhibit variable growth patterns, but there is no standardized characterization of their size relative to anatomical location. Although keloids are generally classified as a single disease entity, significant variations in their morphological and histological features influence treatment choices and therapeutic outcomes. Keloid phenotypes are characterized as sessile vs. nodular and fresh vs. mature.^8^

Despite the recognition that keloids in African people are aggressive, with extensive clinical variations and challenging treatment outcomes, most studies on the clinical and histological characteristics of keloids are based on non-African populations. This has resulted in a significant knowledge gap that fails to account for the unique presentations and pathological features observed in sub-Saharan African populations.

Understanding the clinical and histopathological features of keloids is critical to elucidating their progression and treatment response, particularly in African populations. Addressing this knowledge gap is essential for guiding future research, designing relevant therapies and conducting effective clinical trials.

## Materials and methods

### Study design and setting

This cross-sectional study, consisting of clinical and laboratory-based components, was conducted at Uganda's two largest plastic surgery clinics: the Mulago National Referral and Teaching Hospital (Plastic Surgery Department) and the Kirruddu National Referral and Teaching Hospital (Plastic Surgery Department). Both hospitals serve as national referral centres, receiving patients from across the country. As public institutions, they provide free healthcare, making their services accessible to all Ugandans.

The Plastic Surgery Department at Kirruddu operates a daily clinic, while Mulago runs a weekly outpatient clinic. These clinics address various plastic surgical conditions, including keloids. On average, approximately 300 patients attend these clinics monthly, with approximately 30 presenting with keloids. These clinics are staffed by plastic surgeons and fellows in plastic surgery. The fellows are qualified surgeons undergoing advanced specialization in plastic surgery. Following clinical assessment, patients are scheduled for appropriate treatments such as surgical excision, cutaneous radiotherapy or intra­lesional injections with triamcinolone, among other therapies.

Typically, patients are referred from peripheral healthcare units to receive specialized care from plastic surgeons.

### Study population

The target population included all patients with keloids from any region of Uganda. The accessible population comprised patients with keloids who attended the plastic surgery units at either Kiruddu National Referral Hospital or Mulago National Referral Hospital between August 2023 and February 2024. The study population consisted of patients who were enrolled and registered for care in these clinics during the study period.

### Eligibility criteria

#### Inclusion criteria

All patients who were enrolled at either the plastic surgery clinic at Kiruddu National Referral Hospital or Mulago National Referral Hospital and were diagnosed with keloids by a clinician were eligible for inclusion in the study.

#### Exclusion criteria

For the clinical component of the study, no exclusions were applied. However, for the pathological component, patients with infected keloids were excluded because infections introduce cellular changes that could alter the histological appearance of the keloid tissues.

### Sample size

To determine the sample size of this cross-sectional study, the Keish and Leslie formula was used:[*N* = Z^2^*P*(1 – *P*)/*d*^2^]with Z being the z-statistic of z-distribution, *P* the prevalence estimate of a similar study and *d* the precision set at 5% and with a confidence interval of 95%. A study conducted by Huang *et al.* estimated a prevalence rate of keloids of 16% in African populations, specifically in Zaire.^1^ We obtained a sample size of 206.

To determine the sample size for the histological assessment, we looked at two similar studies conducted in Egypt and Russia,^9^ which used sample sizes of 30 and 50, respectively. Given that the laboratory study was purely descriptive and not intended to make any statistical inferences, we used a sample size of 50 participants, similar to that used by Filippova *et al*.^9^

### Sampling strategy

A nonprobability consecutive sampling method was used, with patients recruited on a rolling basis as they presented at the clinics until the target sample size was reached, with half the participants recruited from each hospital.

For the laboratory-based component of the study, enrollment occurred among patients who were scheduled for surgery and attended their appointments. These patients were also selected on a consecutive nonprobability basis until the desired sample size was achieved.

### Study variables

#### Demographic characteristics

The demographic characteristics collected included the participants' age, sex, ethnicity, region of origin, level of education, family history of keloids and age at onset of keloids.

#### Outcome variables

The outcome variables included the duration of keloid symptoms, history of previous symptoms, pre-existing co­morbidities, keloid site, size, multiplicity and morphology. For the histopathological assessment, the epidermal, dermal and cellular characteristics of the keloids were also described.

### Study procedure

Upon obtaining consent to participate in the study, patients were provided with a questionnaire. Research assistants completed the questionnaires, which included gathering demographic data, as well as keloid characteristics such as site, size and shape. The cause of the original trauma was also recorded, as per the questionnaire. Additionally, photographs and measurements of the keloids were taken. Patients scheduled for surgery were followed up, and excised keloid tissue was collected and placed in a 10% formalin solution. The specimens were then sent to the laboratory for histological analysis. For histology, the samples were fixed in 10% formalin for 24 h, after which paraffin blocks were prepared. Standard haematoxylin and eosin staining was performed by a consultant histopathologist at Gammapath Clinical Laboratories in Kampala, Uganda. Specific morphometric calculations were conducted and reported, and micrographs were captured at a scale of 200 µm.

### Statistical analysis

Data were collected and analysed using STATA 14 (StataCorp, College Station, TX, USA). The analysis was primarily descriptive, focusing on summarizing the clinical and histopathological characteristics of keloids in the study population. Descriptive statistics were calculated for categorical variables, including proportions and corresponding variables for demographics and clinical features. For continuous variables, means and their standard deviations were reported. Additionally, data were presented in tabular formats to enhance clarity and facilitate interpretation. No inferential statistical tests were conducted, as the study's primary objective was to descriptively document the observed findings rather than to establish causal relationships or associations.

## Results

### Demographic characteristics of study participants

A total of 231 participants were recruited for the study and a summary of their demographic characteristics is presented in Table 1.

**Table 1:** Participant demographics (*n* = 231)

Variable	*n* (%)
Age (years), mean (SD)	29.0 (15.9)
Sex	
Female	148 (64.1)
Male	83 (35.9)
Marital status	
Single	138 (59.7)
Married	86 (37.2)
Divorce	7 (3.0)
Number of children (*n* = 187)	
0	62 (33.2)
<2	42 (22.4)
>2	83 (44.4)
Employment (*n* = 179)	
Yes	108 (60.3)
No	71 (39.7)
Level of education (*n* = 217)	
None	15 (6.9)
Primary	66 (30.4)
O-level	58 (26.7)
A-level	14 (6.5)
Tertiary/diploma	28 (12.9)
University	36 (16.6)
Region of origin (*n* = 210)	
Central Uganda	133 (63.3)
Eastern Uganda	32 (15.2)
Northern Uganda	7 (3.3)
West Nile Uganda	5 (2.4)
Western Uganda	33 (15.7)
Monthly income (UGX) (*n* = 83)	
<500 000	47 (56.6)
500 000–1 000 000	28 (33.7)
>1 000 000	8 (9.6)

Data are presented as *n* (%) unless otherwise stated. UGX, Ugandan shillings.

Of the 231 participants, 210 registered their district of residence. The majority, 133 (63.3%) resided in the central region of Uganda, with 42 (20.0%) living in Kampala and 32 (15.2%) in Wakiso. A large majority of the participants (*n* = 190/231; 82.3%) identified as Bantu (Table 2), with the most common tribe being the Baganda (Table 2).

**Table 2:** Ethnicity and tribe of study participants (*n* = 231)

	*n* (%)
Ethnicity	
Bantu	190 (82.3%)
Nilotic	18 (7.8)
Sudanic	9 (3.9)
Non-Ugandan	14 (6.1)
Tribe	
Baganda	97 (42.0)
Nkole	28 (12.1)
Soga	17 (7.4)
Nyoro	11 (4.8)
Other^a^	78 (33.8)

^a^Other encompasses the other 52 Ugandan tribes.

**Table 3:** Clinical characteristics of keloids (*n* = 231)

Characteristic	*n* (%)
Site	
Head	96 (41.6)
Neck	9 (3.9)
Trunk	55 (23.8)
Upper limbs	16 (6.9)
Lower limbs	15 (6.5)
Genitalia	5 (2.2)
Multiple sites	35 (15.2)
Head and neck keloids (*n* = 105)	
Ears	58 (55.2)
Face	29 (27.6)
Neck	9 (8.6)
Scalp	9 (8.6)
Trunk keloids (*n* = 55)	
Chest	27 (49.1)
Breast	8 (14.5)
Back	14 (25.5)
Abdomen	6 (10.9)
Number of keloids	
1	97 (42.0)
2	61 (26.4)
3	8 (3.5)
4	7 (3.0)
5	4 (1.7)
>5	54 (23.4)
Keloid morphology	
Sessile	116 (50.2)
Single nodule	37 (16.0)
Multinodular	21 (9.1)
Pedunculated	26 (11.3)
Invasive/infiltrating	29 (12.6)
Other	2 (0.9)
Keloid size, mean (SD)	
Surface area (cm^2^), *n* = 79	27.77 (34.89)
Height (cm), *n* = 84	0.98 (1.09)
Volume (cm^3^), *n* = 62	18.95 (52.28)
Data are presented as *n* (%) unless otherwise stated.	

### Keloid phenotypes

Broadly, keloids were classified into two groups of either sessile or nodular. Of these two subgroups, 50.2% (*n* = 116/231) of all the keloids were sessile in nature (see Table 3).

#### Sessile keloids

**Figure 1:**
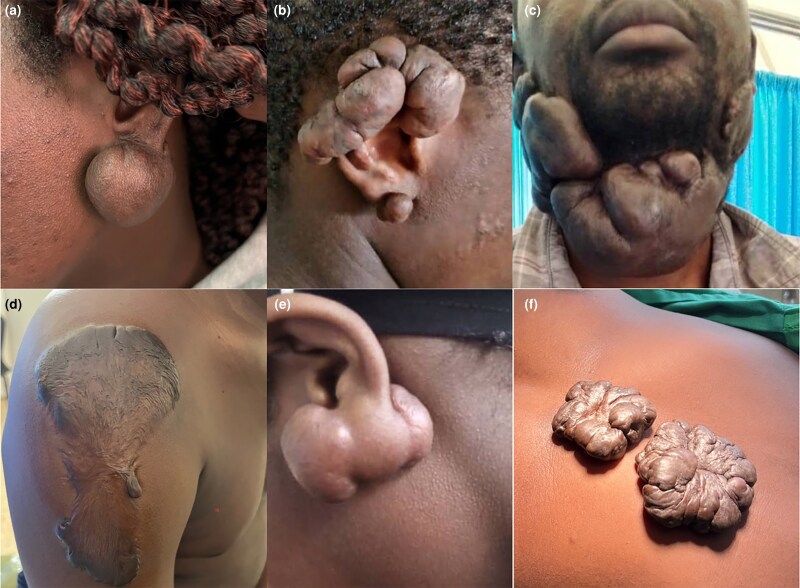
Different keloid phenotypes. (a) A single nodular keloid. (b) A multinodular keloid. (c) A massive raised keloid. (d) A superficial spreading keloid. (e) An invasive keloid. (f) A florid keloid.

**Figure 2:**
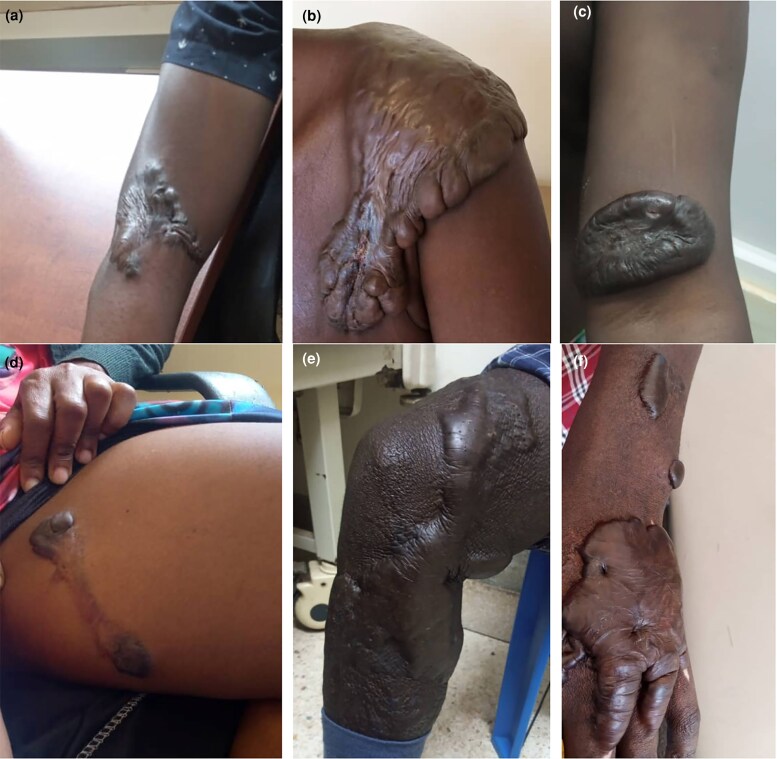
Limb keloids. (a–c) Upper limb keloids. (d) Sessile keloids. (e, f) Keloids crossing joint creases.

**Figure 3:**
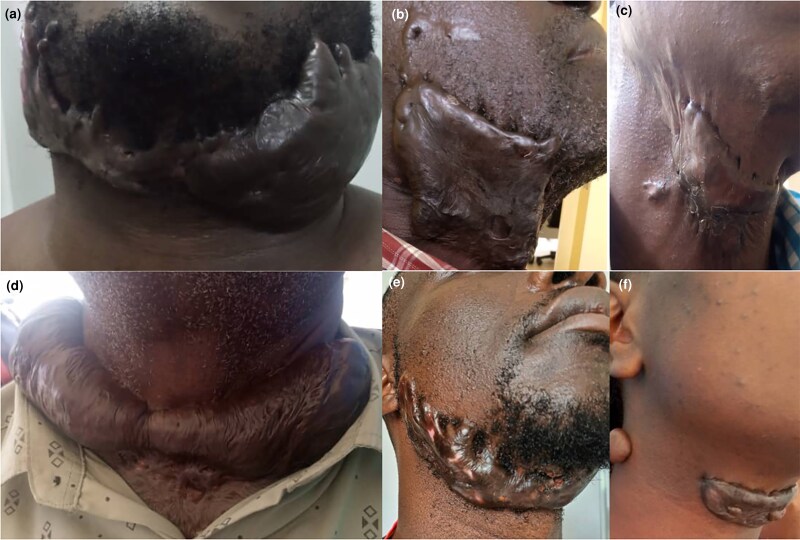
Keloids of the neck. (a, d, f) Homogenously raised/bulging keloids. (b, c, e) Contractures arising from keloids.

**Figure 4:**
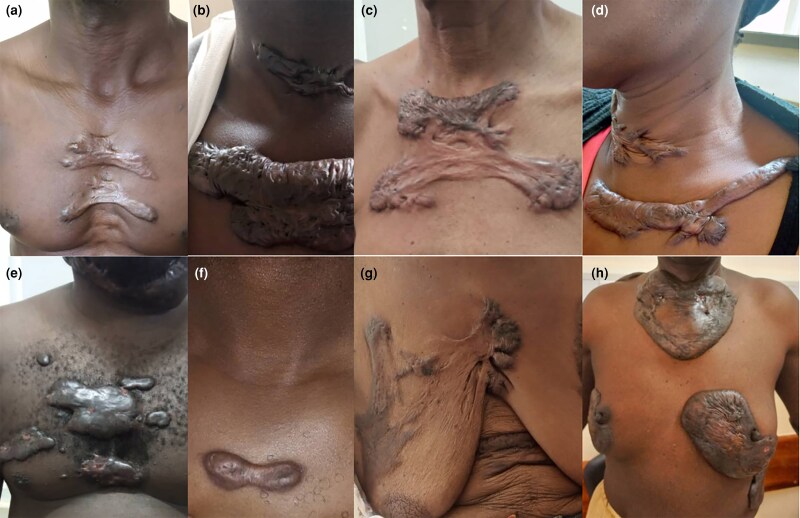
Keloids of the chest. (a, c, g) Superficial spreading flat keloids. (b, d, e, h) Bulging sessile keloids.

Sessile keloids took on either a flat/superficial spreading (Figures 1d, 2d) or raised/bulging sessile form (Figures 1a–c, f, 3b, 4h).

With regard to superficial spreading/flat sessile keloids, the keloids had a flat central area (see Figure 2b, d) that was either thinned out (Figure 2d) or with normal thickness (Figure 1d), and either had similar pigmentation as the skin or was variably hypopigmented (Figure 2b, c). These also had a variably raised and thickened margin/peripheral edge that was also hyperpigmented. Notably, these superficial spreading lesions occurred on the trunk and limbs along the longitudinal axis, following the lines of growth and none occurred along the creases or joint areas.

The raised/bulging sessile keloids consisted of homogeneously raised, firm keloid tissue with no distinct peripheral or central zones (Figures 3a, b and 4a, f). In some of these keloids, the central zone was thicker than the peripheral zone (see Figure 4e, h), in contrast to superficial spreading keloids, which had a thinned-out central zone (see Figure 4c, g). The whole keloid was homogeneous in thickness, consistency and pigmentation. These were observed in joint crease areas (Figures 1e and 3b, c), the neck (Figure 3a, d–f), and were observed in multiple keloids, unlike super­ficial spreading keloids (see Figure 4e, h).

#### Nodular keloids

**Figure 5:**
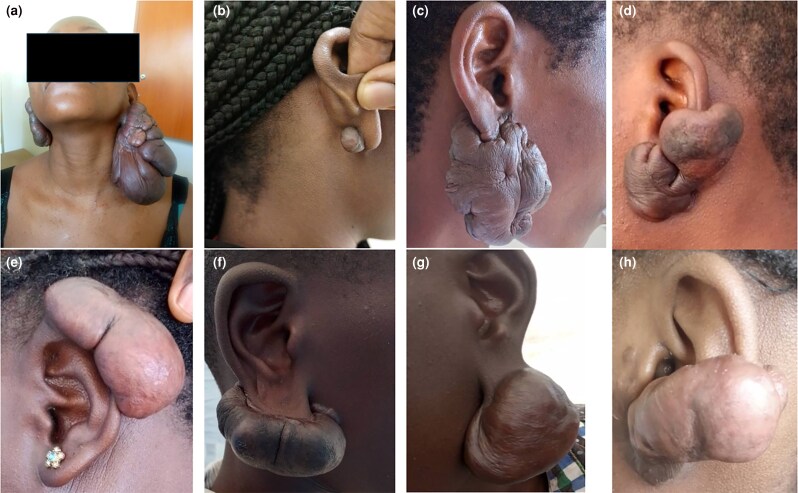
Morphological characteristics of ear keloids. (a) Multinodular bilateral ear keloids. (b) Single nodular. (c) Florid type. (e) Sessile.(f, g) Penduculated keloids. (d, h) Invasive keloids.

These constituted 48.9% (*n* = 113/231) of all the keloids and there were four phenotypes: single nodular (Figure 1a), multinodular (florid) (Figure 1f), pedunculated (Figure 5g) and invasive, as seen in Figure 1. A characteristic of the nodular phenotype was that the keloid base (the part of the keloid in contact with the skin) was not wider than the widest diameter of the keloid.

The single nodular type consisted of a spherical or ovoid keloid attached to the base of the origin. These characteristically had a smooth, shiny overlying skin with no adnexae (hair) and mainly occurred on the ear pinnae.

The multinodular type consisted of more than one nodular keloid, with the nodules described as coalescing or fusing at their bases to varying degrees of fusion. The pedunculated keloids were a variant of the single nodular type but had no basal attachment to the underlying tissues, with the keloid being purely suspended by a stalk of normal skin. In these cases, the keloid and the organ or structure of origin were two distinct entities, merely connected by the skin.

The invasive subtype of the nodular type demonstrated a more distinct and aggressive phenotype, with the keloid – despite arising from the skin invading the underlying structure – completely distorting/disrupting the anatomical integrity of the structure. If it was on the pinna, as shown in Figures 1(c) and 5(d, h), the pinna was destroyed beyond surgical salvage. In areas where these keloids crossed joint areas, especially in the neck, they often caused debilitating contractures (see Figure 3c).

### Clinical characteristics of the keloids

The most frequent locations for keloid formation were the head (including the face, ears and scalp) and the trunk, with 96 (41.6%) and 55 (23.8%) cases, respectively. The rarest site for keloid development was the genitalia, with 5 (2.2) reported cases. Interestingly, more than half of participants (*n* = 134/231; 58.0%) had more than one keloid.

#### Head and neck keloids

One hundred and five participants had isolated head or neck keloids without multiple keloids in other sites. The head was divided into the face, scalp and ears. Although keloids were described in more specific areas of the face such as the jaw area, chin and nasal area, as well more detailed ear locations, for statistical analytical purposes all face keloids were grouped together. More than half (*n* = 58/105; 55.2%) of head and neck keloids involved the ears.

##### Ear keloids

The ears were the most common site for keloid development among the study participants (see Figure 5), with 71 of 231 (30.7%) participants having either a single ear keloid or having ear keloids as one of multiple keloids. The most common site of ear keloids was the lobule, followed by the helix. In some instances, the keloids invaded and distorted the anatomical structure of the ear (see Figure 5h), while in other cases, almost the entire ear was invaded and destroyed by keloids (see Figure 1b). Over 90% of the ear keloids were either nodular or multinodular in nature (see Figure 5).

##### Face, scalp and neck keloids

Facial keloids accounted for 27.6% (*n* = 29/105) of the head and neck keloids, with scalp (*n* = 9/105; 8.6%) and neck keloids (*n* = 9/105; 8.6%) being the least common.

**Figure 6:**
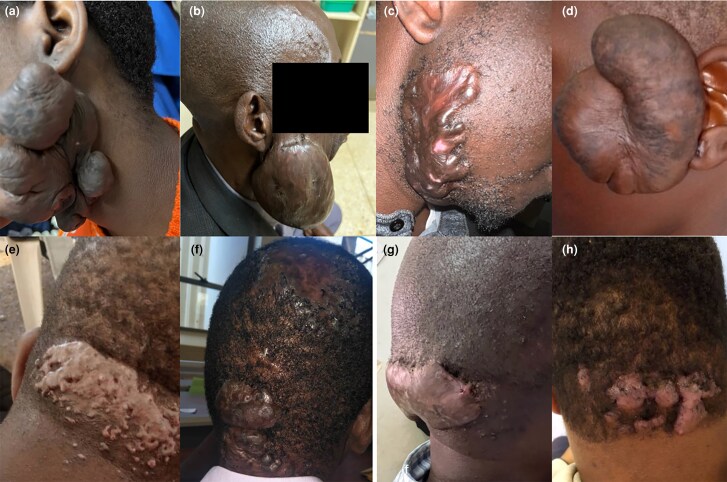
Keloids of the head, face and scalp. (a) Florid type. (b) Large pedunculated. (c, d) Bulging sessile. These keloids are observed to arise from the preauricular submandibular area. (e–h) Occipatal keloids (e) Acneiform type. (f–h) Sessile types.

On the face, the jaw was the most affected area, followed by the chin, while the brow/forehead was the least affected. Scalp keloids were most frequent in the occipital region, followed by the sagittal area, typically presenting with a nodular pattern; occasionally, they appeared in an acneiform manner (see Figure 6e). Neck keloids were larger, more debilitating and often associated with contractures, primarily arising from the submental area of the entire neck (see Figure 3). These keloids frequently co-occurred with others and were firm, infiltrating and restricted neck mobility. Interestingly, keloids were absent on the back of the neck.

#### Keloids of the trunk

**Figure 7:**
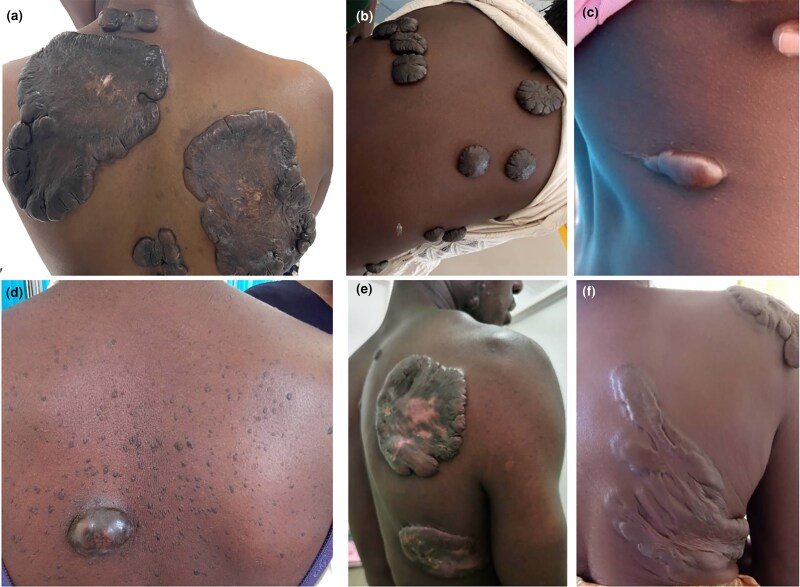
Keloids of the back. (a, e, f) Large sessile keloids. (b) Florid multiple keloids. (c, d) Single sessile keloid.

**Figure 8:**
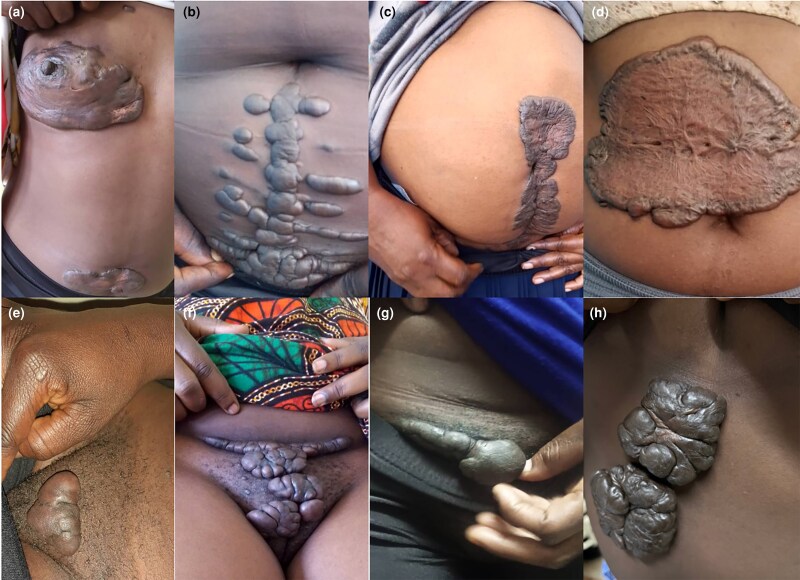
Keloids of the abdomen and genitalia. (a) Breast keloid. (b, c) Postoperative keloids. (d) Keloid following a burn. (e–g) Keloids following shaving. (h) Keloid following a trivial trauma.

Nearly half (*n* = 27/55; 49.1%) of all trunk keloids occurred on the chest, more specifically in the sternal area (see Figure 4). These were distinctively sessile in most cases, while in others, they were bulky, florid and invasive. Similarly, for the back and abdomen, there was a tendency for superficial spreading keloids (see Figure 7). Characteristically, for the abdomen, keloids tended to occur due to postoperative trauma (see Figure 8).

#### Limb keloids

Limb keloids contributed 13.4% of all keloids (*n* = 31/231), with approximately half occurring in either upper or lower limbs. These were predominantly flat and, interestingly, spared the joint areas (see Figure 2), and rarely caused contractures.

### Histopathological characteristics of keloids

Thirty-five of 231 keloids were analysed. A qualitative descriptive approach was used to describe the results, and data collection continued until saturation was reached (i.e. no new information was obtained from the histopathology results). A collective impression of the different histological findings was then made in comparison to normal skin. Haematoxylin and eosin-stained, paraffin-embedded tissue sections were assessed for all keloid tissues. Histological sections were examined under an ICC50E microscope (Leica, Wetzlar, Germany). Notably, all specimens consisted of two main areas: the epidermis and the dermis. The dermis was further divided into the reticular and papillary dermis.

### Epidermis

Overall, the epidermal architecture of the keloids was largely preserved, appearing similarly to the epidermis of the adjacent normal skin.

**Figure 9:**
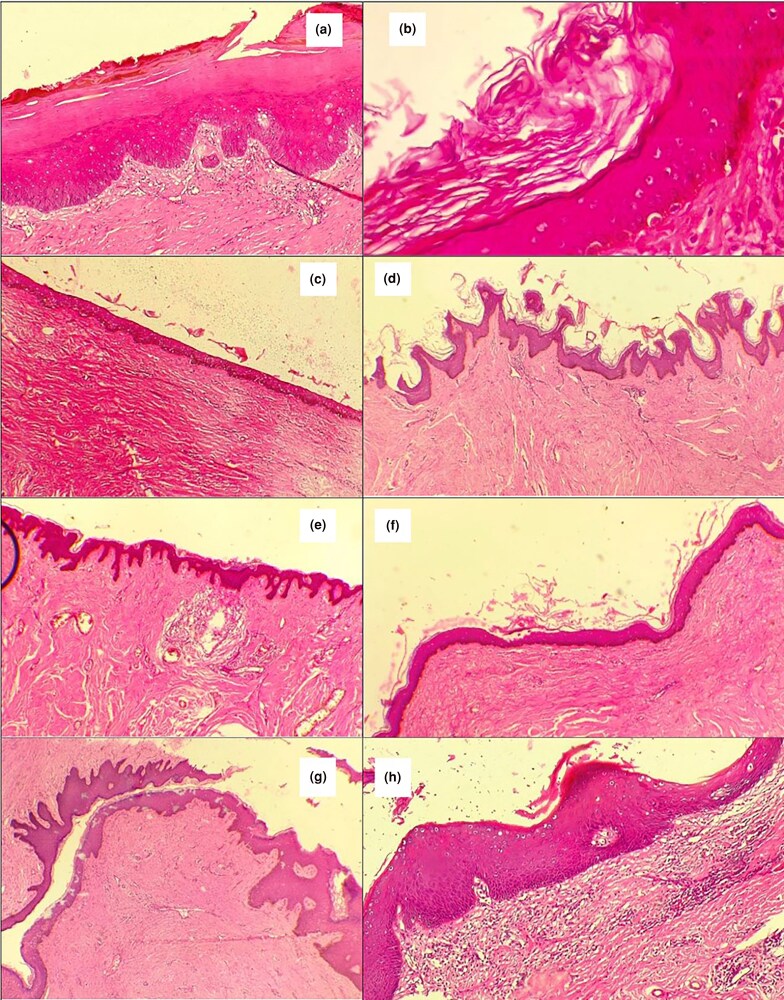
Epidermal characteristics of keloid tissue. (a) Keratin layer of epidermis. (b) Keratinocytes. (c) Thin flat epidermis. (d, e) Prominent epidermal rete ridges. (f) Bulging epidermis. (g) Tongue-like epidermal ridges. (h) Intact dermal–epidermal junction. Haematoxylin and eosin, × 400 magnification.

The deposition of keratin (hyperkeratosis) was increased in 14 of 35 (40%) samples (40%; see Figure 9a). The keratinocytes were distinct and clearly defined in all but one case.

Epidermal thickness was variable, with 13 of 35 (37%) samples showing increased thickness, while normal and atrophic thickness were observed in 11 cases each (31%; see Figure 9). In 21 of 35 (60%) cases, the epidermis was flattened, while in the remaining cases, it was bulging. The epidermal rete ridges were prominent in 11 of 35 (31%) specimens, while more than half (*n* = 18/35; 51%) appeared normal. In 12 of 35 specimens (34%), prominent advancing tongue-like epidermal ridges extend into the dermis (see Figure 9g).

The dermal–epidermal junction was intact and preserved in all specimens. There was little noticeable cellular infiltration into the epidermis, indicating that the disease process was essentially not epidermal in origin.

### Dermis

Overall, significant alterations were seen in the dermal characteristics of all 35 specimens. The dermis consists of the fibrous component composed of collagen and an extracellular ground substance containing various substances, including proteoglycans, hyaluronic acid and glycoproteins. The dermis also comprises different cell types, including mast cells, macrophages and fibroblasts among others. The most superficial aspect of the dermis is the papillary dermis, while the reticular dermis lies deeper.^10^

#### Dermal thickness

**Figure 10:**
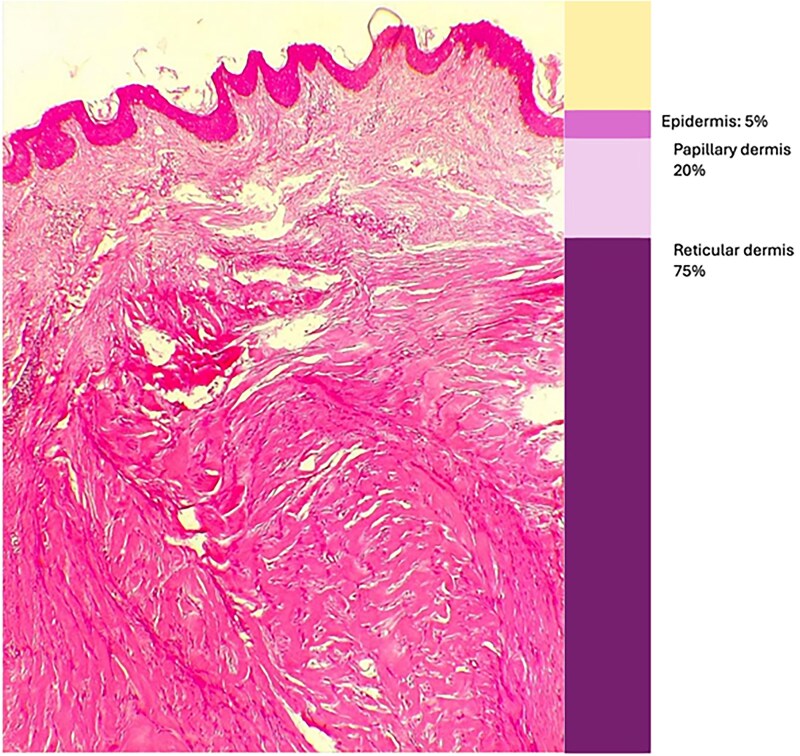
Dermal thickness in keloids. Representative example. Haematoxylin and eosin, × 400 magnification.

Overall, the reticular dermis was notably thicker, accounting for 75% of the total keloid thickness across all specimens (see Figure 10).

#### Dermal cellular infiltrate

**Figure 11:**
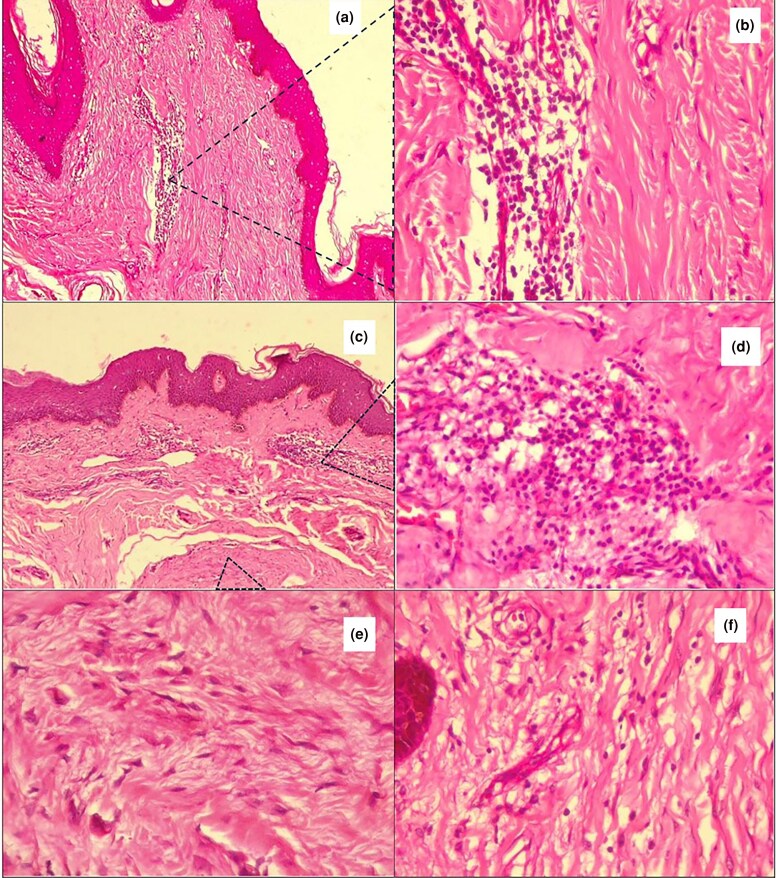
Cellular infiltrate into the keloid dermis. (a–d) Lympocyctic cellular infiltration. (e) Fibroblasts. (f) Macrophages. Haematoxylin and eosin, × 400 magnification.

Analysis revealed a consistent pattern of low-grade chronic inflammation, with increased dermal cellular infiltration observed in 28 of 34 (82%) samples (Figure 11). The degree of cellular infiltrate was markedly increased in 16 of 34 (47%) specimens. The predominant inflammatory cells were mature lymphocytes, found in 26 of 33 (79%) of samples. Lymphocytes were identified as small, round cells with deeply basophilic (deep blue) nuclei. Their nuclei, which are typically round, occupied the majority of the cell, with a high nucleus-to-cytoplasm ratio. These appeared primarily as small, mature lymphocytes, with no evidence of reactive or atypical lymphoid cells, indicating a non-neoplastic process. However, macrophages appeared as larger, more irregular cells with a deeply basophilic nuclei and more abundant pink cytoplasm. Neutrophils, which are typically associated with acute inflammation, were notably absent in the specimens.

The cellular infiltrate was unevenly distributed throughout the keloid tissue. There was a predominance of cellular infiltration in the papillary dermis, with over 90% of the cells being found therein. Similarly, the cellular infiltrate was more prevalent in the peripheral aspects of the keloids while the central areas were predominated by dense collagen hyalinized bundles. The most distinctive cells noted were the lymphocytes (mononuclear lymphocytes), fibroblasts and intravascular red blood cells. There was marked perivascular lymphocyte infiltration in 20 of 35 (57%) specimens.

Fibroblasts appeared as spindle-shaped, elongated cells with oval, basophilic nuclei (see Figure 11e), and were arranged in haphazard, less predictable patterns, often parallel to collagen fibres in the keloids. These cells were moderately increased in 16 of 21 (76%) specimens, and these were more predominant in the reticular dermis than the papillary dermis.

**Figure 12:**
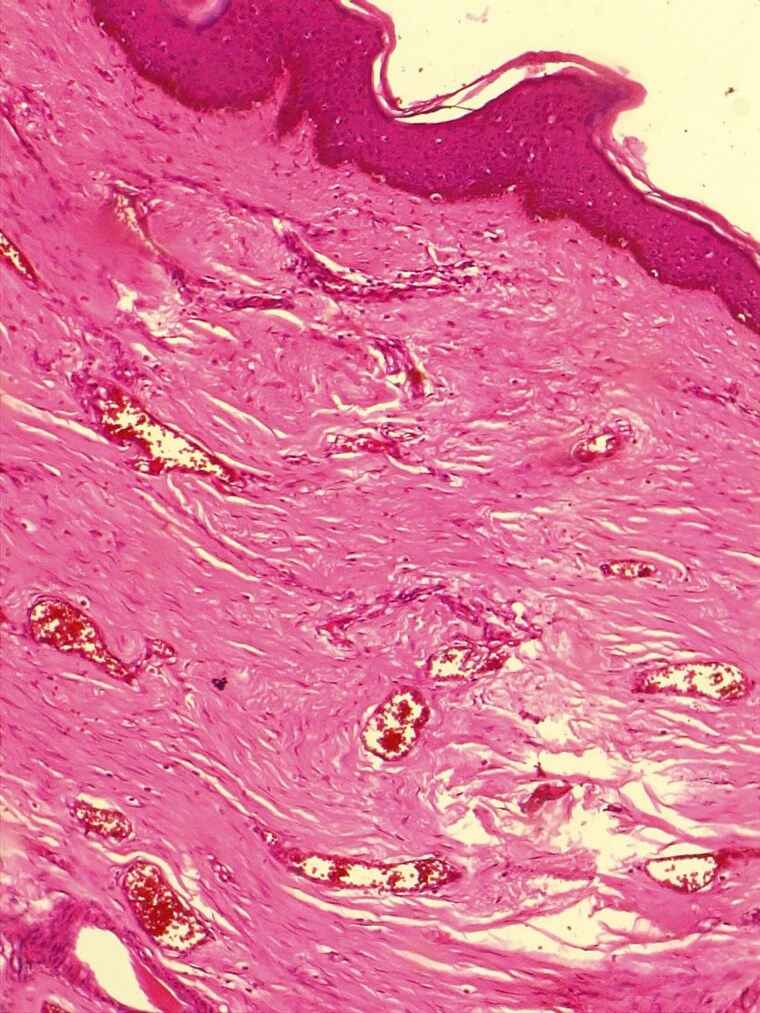
Increased dermal vascularity. Haematoxylin and eosin, × 400 magnification.

#### Dermal vascularity

Twenty-two of 32 (69%) specimens showed increased vascularity, with the majority of dermal vasculature occurring in the papillary dermis (Figure 12).

### Collagen characteristics

**Figure 13:**
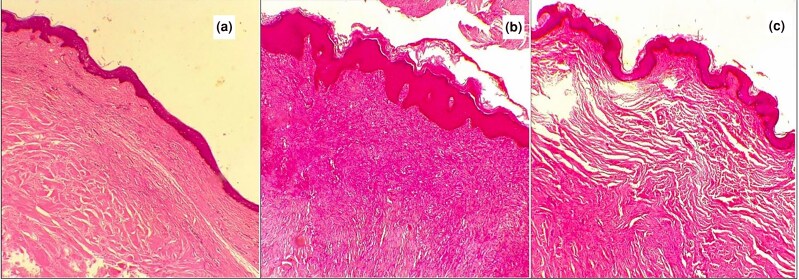
Papillary dermis characteristics. (a) Papillary dermis with parallel collagen. (b) Compact perpendicular collagen. (c) Wavy pattern of collagen. Haematoxylin and eosin, × 400 magnification.

In the papillary dermis, collagen was more compact, organized into small, well-oriented fibres, forming a wavy pattern in 16 of 34 (47%) specimens (Figure 13). The patterns were either parallel to the epidermis (see Figure 13a) or perpendicular (see Figure 13b).

**Figure 14:**
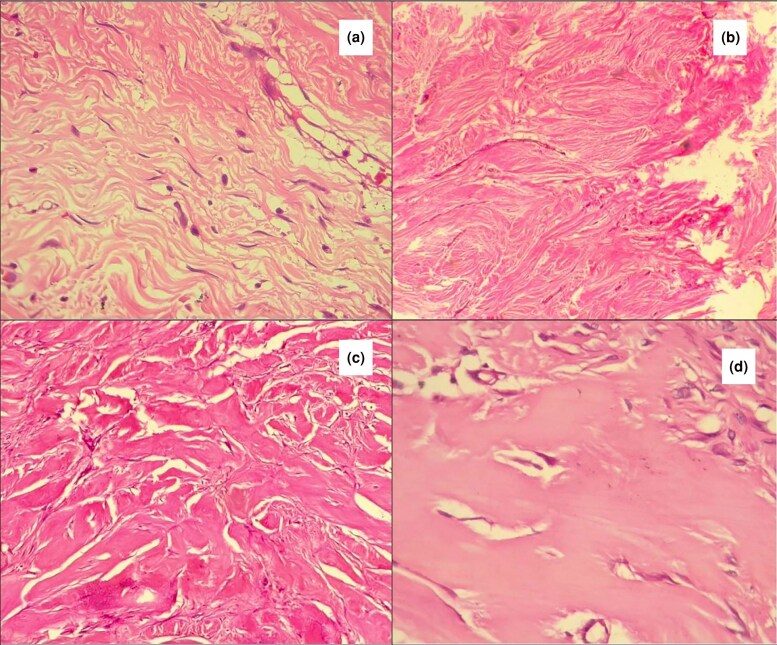
Collagen characteristics. (a) Cellular infiltrate in reticular dermis. (b, c) Disorganized haphazard collagen fibres. (d) Thickened hyalinized collagen bundles. Haematoxylin and eosin, × 400 magnification.

In contrast, the collagen fibres in the reticular dermis had a more haphazard appearance (see Figure 14), becoming more prominent and larger further away from the papillo-­reticular boundary (see Figure 14c).

#### Collagen fibre density

Notably, the density of collagen fibres in the ­papillary dermis remained smaller than in the ­reticular dermis. However, the density of the collagen fibres in the reticular dermis was markedly increased in all specimens, characterized by thick eosinophilic bundles that occupied a significant portion of the reticular dermis.

#### Collagen fibre orientation

In all 35 specimens, there was evidence of disorganized and aberrant collagen fibre orientation, unlike the typical parallel arrangement seen in normal skin dermis. These haphazard orientation patterns were difficult to classify, with some forming whorl patterns and others forming crisscrossing patterns. The collagen fibres were thickened and hyalinized in 24 of 35 (69%), creating a dense matrix of tissue with notably reduced fibroblasts in the surrounding stroma. The collagen fibres had a parallel orientation in 17 of 34 (50%) specimens, while the rest exhibited mixed patterns.

#### Collagen fibre arrangement

The collagen was densely/closely packed in 18 of 34 (53%) of specimens, with 14 of 34 (41%) specimens having loosely packed collagen and the remaining 2 of 34 (6%) specimens exhibiting normally packed collagen. Among the compactly arranged specimen, collagen fibres were organized into dense, compact bundles, forming nodular aggregates or thick sheets that extended through the entire dermal layer. This arrangement obliterated the typical dermal architecture in 20 of 34 (59%) specimens.

#### Hyalinization of the collagen

The papillary dermis exhibited hyalinized collagen in 24 of 34 (71%) specimens. Hyalinized collagen appeared as homogenous, glassy, eosinophilic bundles, indicating a dense, rigid structure. Hyalinization was more prominent deeper into the dermis. Within the hyalinized bundles, there was loss of the fibrillar pattern.

#### Papilloreticular boundary

**Figure 15:**
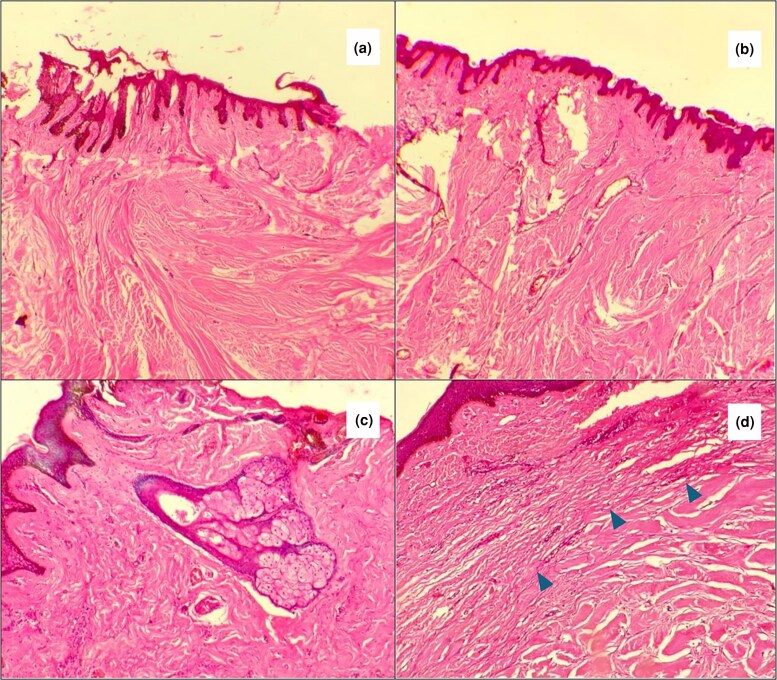
Collagen fibre orientation and patterns. (a, b) Loss of the papilloreticular boundary. (c) Sebaceous glands. (d) Prominent and distinct papilloreticular boundary. Haematoxylin and eosin, × 400 magnification.

The papilloreticular boundary remained preserved in the majority of specimens (*n* = 32/34; 94%) (see Figure 15d). Where it was indistinct (see Figure 15a,b), this was due to the excessive collagen deposition and the abnormal fibre arrangement of the reticular dermis invading the papillary dermis. In some instances, the reticular dermis was compacted and thinned out along the epidermis.

#### Dermal adnexae

Dermal adnexae were reduced in the specimens, occurring in only 10 of 32 (31%) specimens. These included sweat glands, sebaceous glands (see Figure 15c) and hair follicles.

## Discussion

The demographic profile of the study participants provides valuable insight in the epidemiology of the keloids. In this study, the majority of the participants were young adults with a mean (SD) age of 29.04 (15.94) years, reflecting the higher prevalence of keloids in younger age groups. The findings align with similar studies that highlight keloids as a disease that primarily affects young adults.^11–14^

There was a female-to-male ratio of 1.8. This finding is consistent with other studies, suggesting a higher prevalence of keloids among women.^11,15,16^ This could not only imply that women are more likely to be affected than men, but it could also suggest that women are more likely to seek healthcare.

There is global recognition of the impact of ethnicity on keloid development.^1,4,17^ Despite the knowledge that there is a higher prevalence of keloids among Black and African populations,^18,19^ no further assessment of African ethnicities has previously been undertaken. More than 80% of participants in this study identified as Bantu. This may be explained by two factors: firstly, approximately 70% of Uganda’s population is of Bantu ethnicity; secondly, the distribution of other ethnicities in the study reflected their proportions in the general population.^20,21^ This implies that the findings were representative of the ethnicities and not biased toward Bantu.

Forty-two per cent (*n* = 97/231) of the study participants belonged to the Baganda tribe, making people in this tribe the most affected by keloids. People belonging to the Banyankole and Basoga tribes were the next most commonly affected by keloids. The tribal distribution correlates with the national population distribution as per the Uganda National Census,^21,22^ with the order of tribes by population following a similar pattern. The Baganda tribe is one of the predominant tribes in Uganda in terms of numbers. The fact that the study sites were in central Uganda, where the Baganda is the predominant tribe, also explains the relatively high number of study participants from this tribe. The inclusion of participants from nearly all the tribes in Uganda suggests that keloids affect nearly all ethnicities.

The fact that over 80% of patients had keloids for more than 1 year, with nearly half experiencing the condition for over 5 years, highlights the chronic nature of keloids and underscores the long-term challenges they pose for affected individuals.^1^

In our study, the most affected site was the head, followed by the trunk. This is consistent with other studies done in Africa.^8,23^ Notably, the genitalia, which are rarely reported in keloid cases, were affected in 2.2% (*n* = 5/231) of patients. These findings are consistent with previous studies,^7,23,24^ which have identified the head and ears as the most common sites of keloid formation. With regard to the ears, this is likely due to the cosmetic practice of ear piercings. On the trunk, the chest was the most affected region, followed by the back. Furthermore, a considerable number of patients had multiple keloids, suggesting a systemic predisposition to keloid formation.

The study identified a range of keloid phenotypes, providing insight into the variability in clinical presentation and response to therapy.^25^

While the majority of studies, with few exceptions, do not emphasize variations in keloid phenotypes and their associated response to therapy,^25,26^ it is generally understood that keloid phenotypes significantly affect treatment outcomes.^25^

The most common phenotype observed in our study was the sessile type, found in 50.2% (*n* = 116/231) of patients, particularly on larger areas such as the trunk and chest.

The single nodular type, a well circumscribed and elevated lesion, was the second most common phenotype, observed in 16.0% (*n* = 37/231) of cases. These keloids, often seen on the ears and face,^27^ were found to be localized, singular and smaller than the sessile type.^27^

Multinodular keloids, characterized by the presence of multiple nodules within the same lesion or in close proximity, was less common but noted in 9.1% (*n* = 21/231) of the participants. Multiple keloids were more prevalent among participants with multinodular keloids. This phenotype often occurred in areas of repetitive trauma such as the upper arms, abdomen and neck.

The invasive/infiltrative type was another observed phenotype. This type is distinct from the sessile keloids due to its tendency to invade deeper tissues, characterized by the distortion of the affected area and the ability to cross natural skin creases.

Several potential mechanisms have been proposed to explain the variability in keloid phenotypes. Sessile keloids typically develop in areas of high tension, where the tension facilitates horizontal spread. In contrast, nodular keloids often form in regions without significant tension, leading to their characteristic raised and nodular appearance. Multi-nodular keloids are thought to arise from repeated cycles of localized trauma, including multiple therapeutic interventions, which contribute to their recurrence and complex morphology.

There is limited literature on the average size of keloids, and no standard measurement technique exists. Therefore, it was difficult to compare keloid sizes with those reported in other studies. In our participants, keloids were generally large relative to the area of origin (e.g. the ear and chest), with a mean (SD) area of 27.77 (34.89) cm^2^. The size of the keloid may be attributed to aggressive and rapid growth or delayed/ineffective therapy.

Key insights from the epidemiological and phenotypic characteristics of the keloids include their tendency to occur in younger individuals with an almost equal sex predisposition. This implies that there needs to be special emphasis placed on keloid care in this demographic. In Uganda, for example, keloids remain a relatively neglected disease, with little-to-no special consideration in strategic healthcare planning.

Our study findings emphasize that keloids are primarily a disease of the dermis, characterized by abnormal and excessive deposition of collagen as a result of a chronic inflammatory process associated with abnormal wound healing.^17,28^

The increased dermal cellular infiltrate is consistent with a low-grade, immune-modulated chronic inflammatory process, which is consistent with existing literature.^28–30^ The predominance of mature lymphocytes further indicates that this is a low-grade autoimmune process,^30^ unlike the presence of immature, large lymphocytes as would be seen in cutaneous malignancies. This confirms that keloids are purely a benign disease and carry little-to-no risk of malignant transformation.^31^

The aberrant, disorganized collagen fibres, with no particularly predictable orientation, formed the hallmark of the keloid dermis in our specimens. As described in other studies,^32–35^ these fibres represent the distinctive dermal characteristics of keloids, with some studies using the term ‘keloid collagen’^32,33^ to refer to this bizarre pattern of collagen deposition. Overall, the dermal characteristics of keloids among African participants did not differ significantly from those in a non-African population.^34^ Similarly, hyalinization appeared to be a distinctive characteristic in the keloids, similarly to White populations.^1,33,34^

In the sub-Saharan African context, there were assumptions that keloids could demonstrate more aggressive histological characteristics with atypical findings. However, this study provides insight into the fact that, despite the aggressive clinical nature of these keloids, their histopathological architecture essentially remains unchanged.

This study provides a benchmark for comparing keloid characteristics across different populations. Few studies have extensively profiled the phenotypic and histological characteristics of keloids in African populations. Anecdotal statements, often made in passing, suggest that African people generally have more aggressive keloid phenotypes, with a tendency toward atypical characteristics. However, these claims are routinely quoted without supporting ­evidence.

This study has therefore attempted to establish the baseline characteristics of keloids in this population group and forms a basis for further inquiry into understanding keloids in sub-Saharan African populations.

Owing to the lack of literature on keloids in Uganda and sub-Saharan Africa, keloids are often neglected and rarely prioritized in strategic healthcare planning. The finding of how severe the disease is and the fact that it occurs in younger adults implies the need for strategic recognition of keloids as a disease process rather than merely a cosmetic problem, as is commonly assumed.

The study was not designed to determine the population prevalence of keloids. However, recruiting participants from a hospital setting inherently limits the inclusion of patients to those who sought medical care at national referral hospitals. This likely excludes individuals with milder forms of keloids who may not perceive the need for treatment or to seek care at national referral hospitals. Consequently, the findings may disproportionately reflect severe cases, potentially overestimating the severity of keloids in our population group.

The recruitment of participants from referral hospitals introduces a referral bias. Typically, these facilities care for patients with more advanced or complicated cases, implying that the study may over-represent individuals with severe keloid presentations. This bias limits the generalizability of the findings to primary- or community-level healthcare settings, where patients with less severe forms of keloids may predominate.

All histopathological specimens were reviewed by a single experienced pathologist, ensuring consistency but introducing the potential for observer bias due to the lack of interobserver validation. This could have affected the reliability and reproducibility of findings. Additionally, prior treatment within 3 months of surgical excision may have influenced the histological characteristics of the keloids, although the study focused solely on describing the state of the tissues postexcision as they were.

As a descriptive study, the research did not aim to establish causal relations between clinical and histopathological findings. This limits our ability to provide direct clinical correlations or implications for treatment effectiveness. While this was outside the scope of the current research, the baseline data generated serve as a foundation for future analytical and interventional studies to explore these relationships.

The study findings may not fully capture geographical diversity, as participants were recruited from national referral centres in central Uganda. Regional differences in healthcare access, cultural practices and environmental factors could influence keloid characteristics.

The study did not incorporate molecular and epigenetic analyses, which could have offered deeper insights into the genetic and cellular mechanisms of keloid pathogenesis. This highlights the need for future research integrating molecular approaches to complement the current findings.

Building on the limitations identified in this study, future research should aim to address selection and referral biases by incorporating community-based sampling methods to capture a broader spectrum of keloid severity and prevalence. Longitudinal studies would provide valuable insights into the progression, treatment response and recurrence of keloids, offering a better understanding of cause-and-effect relationships. Molecular and epigenetic analyses are critical next steps to explore the genetic and cellular mechanisms driving keloid pathophysiology. Expanding the geographical scope of research will help uncover any regional variations.

In this descriptive study on the clinical and histopathological characteristics of keloids among sub-Saharan African patients, most affected individuals were young adult women of Bantu ethnicity, who mainly presented with large head and trunk sessile keloids. There was a tendency for multiple keloids, with a quarter of the participants having five or more keloids. The histological characteristics consisted of primarily haphazard excessive deposition of hyalinized collagen, similar to reports in non-African population groups.

These findings provide valuable insights into understanding keloids in the sub-Saharan African population and form a scientific foundation upon which prospective and analytical studies in African populations can be conducted.

## Data Availability

The data underlying this article will be shared on reasonable request to the corresponding author.
